# The safety of Covid-19 mRNA vaccines: a review

**DOI:** 10.1186/s13037-021-00291-9

**Published:** 2021-05-01

**Authors:** Pratibha Anand, Vincent P. Stahel

**Affiliations:** 1 University of Colorado (CU) School of Medicine, 13001 E 17th Place, Aurora, CO 80045 USA; 2 University of Colorado (CU) Boulder Undergraduate Program, Boulder, CO 80309 USA

**Keywords:** Coronavirus, COVID-19, SARS-CoV-2, mRNA vaccine, Vaccine safety

## Abstract

The novel coronavirus disease 2019 (COVID-19) has infected more than 100 million people globally within the first year of the pandemic. With a death toll surpassing 500,000 in the United States alone, containing the pandemic is predicated on achieving herd immunity on a global scale. This implies that at least 70-80 % of the population must achieve active immunity against the severe acute respiratory syndrome coronavirus 2 (SARS-CoV-2), either as a result of a previous COVID-19 infection or by vaccination against SARS-CoV-2. In December 2020, the first two vaccines were approved by the FDA through emergency use authorization in the United States. These vaccines are based on the mRNA vaccine platform and were developed by Pfizer/BioNTech and Moderna. Published safety and efficacy trials reported high efficacy rates of 94-95 % after two interval doses, in conjunction with limited side effects and a low rate of adverse reactions. The rapid pace of vaccine development and the uncertainty of potential long-term adverse effects raised some level of hesitation against mRNA vaccines in the global community. A successful vaccination campaign is contingent on widespread access to the vaccine under appropriate storage conditions, deployment of a sufficient number of vaccinators, and the willingness of the population to be vaccinated. Thus, it is important to clarify the objective data related to vaccine safety, including known side effects and potential adverse reactions. The present review was designed to provide an update on the current state of science related to the safety and efficacy of SARS-CoV-2 mRNA vaccines.

## Background

Severe acute respiratory syndrome-2 virus (SARS-CoV-2) represents a highly contagious respiratory virus that is responsible for the current worldwide coronavirus disease 2019 (COVID-19) pandemic [1]. Currently, there have been over 30 million cases of COVID-19 in the United States, with reported deaths of more than half a million people at the time of the drafting of this article (www.cdc.gov). Due to the serious implications of this pandemic, a scientific focus on vaccine development for COVID-19 has been the forefront of a global initiative to combat this virus [2–4].

## History of vaccines

The establishment of vaccines represents a vital instrument in reducing rates of infections and diseases globally [5]. The original premise of vaccination, dating back to the eleventh century, was human exposure to a small amount of a disease, to promote protection and immunity against subsequent exposure of a larger quantity of the same pathogen [6]. This was first recorded in Chinese literature, whereby ingesting small amounts of poison could prevent potential fatality of a larger poison dose [7, 8]. Furthering these basic principles of vaccination, research conducted by Louis Pasteur in the 19th century resulted in the discovery of attenuated pathogens that were inoculated into a subject, in order to prevent a potential ensuing infection when exposed to the same pathogen [9]. A significant aspect of this discovery was the ability to attenuate a pathogen [10]. This provided the ability to inject a pathogen that would result in limited adverse effects, elicit an immune response to develop protection, and prevent contraction of the disease [11].

Vaccine development has persisted into the modern day, and while based on the same principles, the methods of antigen introduction have evolved [12]. The vaccine platform introduced by Pasteur is still highly relevant to current vaccine research, but an array of new vaccine platforms are available in the modern day [13]. Current vaccine platforms include: live-attenuated, inactivated, toxoid, subunit, recombinant, polysaccharide, conjugate, and the most recent platform; mRNA vaccines [14]. All of these mechanisms of vaccination have ultimately derived from the basic principles that were uncovered hundreds of years ago [15]. A summary of historical landmarks in vaccine development is shown in Table 1.

**Table 1:** Historical landmarks in vaccine development

18th Century	19th Century	20th Century (1st half)	20th Century (2nd half)	21st Century
Smallpox	Rabies	Diphtheria Toxoid	Polio	Pneumococcal Conjugates
	Typhoid	Tetanus Toxoid	Measles	Meningococcal Conjugates
	Cholera	Pertussis	Mumps	HPV
	Plague	Tuberculosis	Rubella	Zoster
		Yellow Fever	Anthrax	Rotavirus
		Influenza	Adenovirus	Cholera
		Rickettsia	Tick Encephalitis	Japanese Encephalitis
			Hepatitis B	Pneumococcal Conjugates SARS-CoV-2

## The mRNA vaccine platform

The concept of mRNA vaccines has been scientifically relevant since the early 21st century, however, the development of the Pfizer/BioNTech and Moderna COVID-19 vaccines presents the initial, large scale, application of this type of inoculation [16]. Previous platforms have utilized similar mechanisms of vaccination by exposing a subject to a pathogen, or a specific aspect of a pathogen, such as a sugar or capsid. However, mRNA vaccines provide a novel and alternative approach to providing pathogen immunity [17]. Messenger RNA vaccines provide the genetic code of the pathogen’s relevant antigen. This messenger RNA is then translated by the host to form the relevant protein from the pathogen being studied. In other words, the vaccine provides the cells with a blueprint to construct the protein [18]. This process allows the host to mount an immune response against the constructed foreign protein [18]. The cells then destroy the blueprint, the injected mRNA, following the development of the protein [18]. The half-life of the mRNA is short and remains in human tissues for just a few days [19]. The immune response elicits the production of antibodies, which allows the body to develop a certain degree of immunity against the specific pathogen [20]. A similar immune response would be generated through natural contraction of COVID-19, but with the mRNA vaccine, the body will not be required to endure the actual exposure to the pathogen, while still mounting an immune response [21]. A common misconception is that mRNA vaccines represent a new vaccine platform developed to combat COVID-19. Whereas, mRNA vaccines have been designed and developed for years against other pathogens, such as ebola, zika, rabies, influenza, and cytomegalovirus [22, 23].

**Fig. 1:**
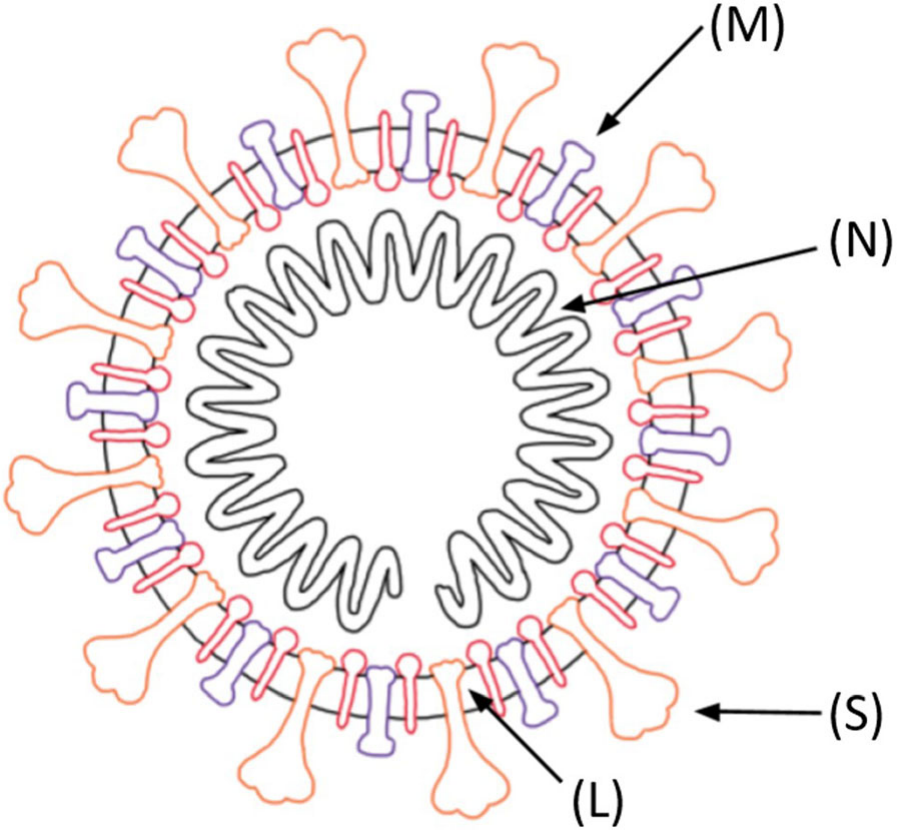
Structure of the SARS-CoV-2 virus. (M) Membrane protein. (N) Nucleocapsid (capsid protein & RNA). (S) Spike protein. (L) Lipid bilayer

**Fig. 2:**
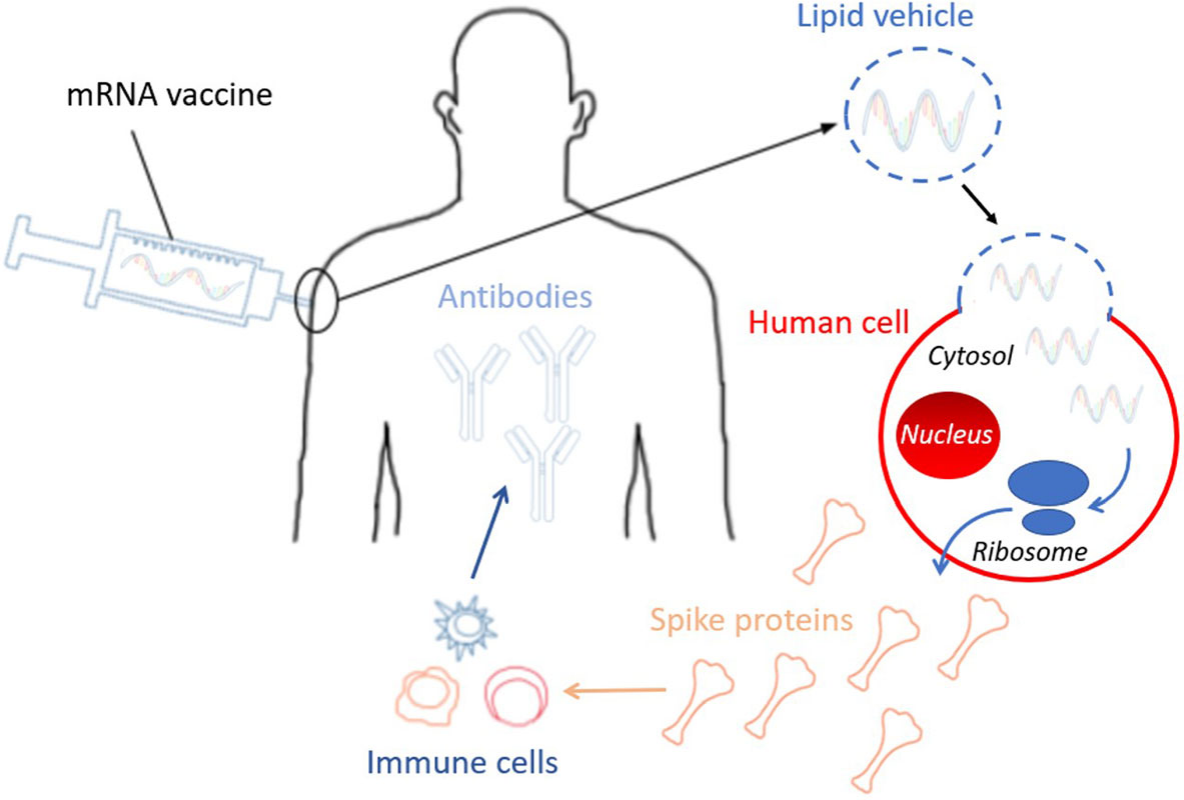
COVID-19 mRNA vaccination mechanism. The mRNA vaccine is injected by intramuscular route, typically into the deltoid muscle. The lipid coat vehicle around the mRNA allows for the vaccine to enter the cytosol of the cell. The ribosomes translate the mRNA into spike proteins. The injected mRNA subsequently degrades. The spike proteins are released from the cell and initiate an adaptive immune response. Through various activation pathways, immune cells mount a cell-mediated and antibody-mediated immunity against the spike protein of the SARS-CoV-2 virus

When specifically applying the mRNA vaccine platform to SARS-CoV-2 (Fig. 1), it is crucial to understand the specific mRNA vaccine mechanism [24, 25]. In the case of SARS-CoV-2, the mRNA provides the genetic blueprint for the spike protein of COVID-19 [26]. Specifically, the vaccine is a lipid nanoparticle-encapsulated mRNA vaccine that encodes the perfusion stabilized full-length spike protein [2]. Lipid nanoparticles – which are the most commonly utilized vectors for *in vivo* RNA delivery – shelter mRNA from degradation, and mediate endocytosis and endosomal escape [27]. Positively charged lipid nanoparticles help bring mRNA to the negatively charged cell membranes, facilitating subsequent cytoplasmic endocytosis. For the mRNA to be transcribed, it must escape both the lipid nanoparticle as well as the endosome [28]. The immune cells then display the spike protein on their surface and break down the instructions to build the spike protein that was provided by the mRNA vaccine (Fig. 2). The immune system recognizes that this protein is foreign and instructs the immune system to develop antibodies against COVID-19 [24]. This mechanism provides the immune system with protection against subsequent infection and bypasses risks associated with injecting the actual pathogen into the body, whether alive or attenuated [5, 7].

## Clinical trials

Development of a vaccine to combat COVID-19 has been of paramount importance since the onset of the pandemic [2]. The scope of this review focuses on the safety and efficacy of the Moderna and Pfizer/ BioNTech mRNA vaccines exclusively. The Pfizer/BioNTech vaccine (BNT162b2) trial reported that the vaccine had 95 % efficacy [29]. The trial enlisted a total of 43,548 adult volunteers, with half of the participants receiving a placebo injection, and the other half receiving the actual vaccine. One hundred seventy people contracted COVID-19 in both groups: 8 of these participants were in the vaccine group, and the other 162 participants were in the placebo group. Ten of the 170 cases were classified as severe, and 9 out of the 10 severe cases were among participants in the placebo group [29].

The Moderna vaccine (mRNA-1273) trial enrolled 30,420 volunteers, with half of the participants received the vaccine, while the other half received the placebo [30]. Of the 15,210 participants in the placebo group, 185 contracted COVID-19 compared to 11 participants that contracted the virus in the vaccine group. These results demonstrated 94.1 % efficacy of the vaccine [30]. At the time of drafting this article, the efficacy of COVID-19 mRNA vaccines against novel mutant strains of SARS-CoV-2 remains unknown and is subject for further investigation.

## Safety considerations

### Common side effects

As of publication of this review, there have been no serious side effects identified in the ongoing phase 3 clinical trials for both the Moderna and Pfzier/BioNTech mRNA vaccines [29, 30]. Mild local side effects including heat, pain, redness, and swelling are more common with the vaccines than with the placebo (normal saline) [29, 30]. Other systemic side effects including fatigue, fever, headache, myalgias, and arthralgias occur more frequently with the vaccine than with placebo, with most occurring within 1 to 2 days following vaccination [29, 30]. Hypersensitivity adverse side effects were equivalently reported in both the placebo and vaccine groups in both trials [31]. As reported, a two-dose regimen of the mRNA vaccines resulted in 94-95 % protection against COVID-19 in people ages 16 and older, and over a median of 2 months, safety of the vaccine was comparable to that of other viral vaccines [29, 30].

### Local reactions

The initial published trials on mRNA vaccines against COVID-19 reported more local reactions in the recipients of the vaccine compared to the control group receiving placebo [29, 30]. The most common local reaction was pain at the injection site within one week of vaccination. The majority of the local reactions were mild-to-moderate in severity and lasted between 24 and 48 h [29, 30]. Across all age groups, less than 1 % of participants reported severe pain and pain of any kind was reported less commonly among participants over the age of 55 years [29, 30].

### Systemic reactions

In the same trials, the younger vaccine recipients (between 16 and 55 years of age) reported systemic events more frequently than their older counterparts (over 55 years of age) [29, 30]. This higher incidence of systemic events may represent a more robust immune response in younger individuals compared to the older population. More side effects were reported following the second vaccine dose compared to the first dose [29, 30]. Following the second dose, fatigue and headache were the most commonly reported side effects. However, these side effects were also reported by a large number of control patients in the placebo group [29, 30]. The incidence of systemic side effects was less than 1 % following the first vaccine dose and less than 2 % following the second dose, with the exception of fatigue (3.8 %) and headaches (2.0 %) [29, 30]. Following the first dose, only 0.2 % of vaccine recipients and 0.1 % of placebo recipients reported fever up to 40 °C. Following the second dose, 0.8 % of vaccine recipients and 0.1 % of placebo recipients reposted fever up to 40 °C. Temperatures above 40 °C were reported by two individuals each in the vaccine and placebo groups. Systemic events including chills and fever resolved within 24 to 48 h post-vaccination [29, 30].

### Adverse events

Adverse events were more commonly reported among vaccine recipients (27 %) compared to the placebo group (12 %) [29, 30]. These ratios are largely attributable to variations in transient reactogenicity events that were reported more frequently in the vaccine group. Related serious adverse events were reported by four vaccine recipients (vaccine administration-related shoulder injury, right axillary lymphadenopathy, paroxysmal ventricular arrhythmia, and right leg paresthesia). Two vaccine recipients died (one from arteriosclerosis, one from cardiac arrest) compared to four fatalities in the placebo group (one from hemorrhagic stroke, one from myocardial infarction, and two from unknown causes). None of the deaths were found by the investigators to be connected to the vaccine or placebo. Furthermore, no COVID-19-associated mortalities were observed, and no halting rules criteria were met during the reporting period. Following administration of the second dose of vaccine, safety monitoring is planned to continue for 2 years [29, 30]. Recent data suggest the incidence of anaphylaxis episodes attributable to the Pfizer/BioNTech vaccine occured in roughly 1:200,000 individuals [29]. This rate lies in sharp contrast to the rate of less than 1 per million doses for most vaccines. The causative antigen and associated mechanisms remain under investigation, although polyethylene glycol has been proposed as the potential culprit [32]. The Pfizer/BioNTech anaphylaxis events have been responsive to epinephrine treatment, although many cases required more than one dose of epinephrine [33]. Both the Pfizer/BioNtech BNT162b2 and Moderna mRNA-1273 vaccines had associated orofacial and oculofacial side effects [34]. These observed adverse events were rare (around 1:1,000) and included facial, labial, and glossal edema [34]. Acute, temporary, unilateral peripheral facial paralysis (Bell’s palsy, an idiopathic palsy of cranial nerve VII) was also reported [34]. This adverse event pertained to individuals who had previously undergone facial cosmetic injections [34] or to people with a known history of Bell’s palsy [35]. A summary of the common side effects, major adverse events and speculated complications is shown in Table 2.

**Table 2:** Side effects and adverse reactions to mRNA vaccines against SARS-CoV-2

Common side effects	Major adverse reactions	Unverified complications
Heat, pain, swelling and erythema at the injection site	Anaphylaxis/anaphylactic shock	Infertility
Fever & chills	Bell’s palsy	Premature childbirth
Fatigue		Autoimmune disease
Headaches		
Decreased appetite		
Myalgia, arthralgia		

## At‐risk populations

### Older adults

The most commonly reported side effects associated with the vaccine among those over the age of 55 years old were chills, headache, injection site pain, fatigue, and myalgias [36]. A higher incidence of local and systemic reactions was reported following the second vaccine dose [29, 30]. Symptoms usually arose within 24 h of the vaccination and resolved quickly. Mild erythema lasting between 5 and 7 days was reported by three participants. Myalgia lasting 5 days that began on day 3 post-vaccination was reported by one participant. Only two systemic adverse events that were classified as severe took place following the second dose: one participant between 56 and 70 years old in the 25-µg dose subgroup reported a fever and another participant over the age of 70 in the 100-µg dose subgroup reported fatigue [37]. Out of the 71 adverse events reported, 17 were considered by the investigators to be associated with the vaccine. All of these adverse events were classified as mild except for one “moderate” case of decreased appetite reported by a participant between 56 and 70 years old in the 25-µg dose subgroup. One severe case of hypoglycemia (glucose level, 50 mg per deciliter; reference range, 65 to 99 mg per deciliter) by a participant between 56 and 70 years old in the 100-µg dose subgroup after fasting and engaging in vigorous exercise. This complication was deemed by the investigators as not being related to the vaccine [37].

### Pregnant women

There is limited data regarding the use of the Pfizer/BioNTech and Moderna COVID-19 mRNA vaccines during pregnancy. The Center for Disease Control (CDC) reports that about 25 % of women of reproductive age (15–49 years of age) hospitalized with COVID-19 between March 1 and August 22, 2020 were pregnant, and that pregnant women tended to require mechanical ventilation more than their nonpregnant counterparts [38]. The CDC also indicates that women infected with COVID-19 during pregnancy are at a greater risk for preterm birth [38]. Among the infants born to SARS-CoV-2 infected women with known gestational, 12.9 % were preterm (< 37 weeks), compared to a national estimate of 10.2 % [39]. Endocrinological, immunological, and physical gestational changes place pregnant women and their fetuses at increased risk for significant complications caused by infectious diseases, which is not unique to COVID-19 [40]. The Pfizer/BioNtech and Moderna COVID-19 mRNA vaccines currently approved through emergency use authorization (EUA) do not utilize an adjuvant and are not live vaccines [29, 30]. Thus, the American College of Obstetricians and Gynecologists (ACOG), and Society for Maternal-Fetal Medicine (SMFM) recommend that these vaccines should not be withheld from pregnant and breastfeeding women [41]. Nevertheless, none of the approved COVID-19 vaccines have been tested for efficacy, immunogenicity, reactogenicity, or safety in pregnant women to date [41]. Results of the Pfizer-BioNtech mRNA BNT162b2 vaccine demonstrate a broad immune response to the vaccine including stimulation of neutralizing antibody responses, stimulation of CD4 + cells, and growth of effector memory CD8 + T cells in men and in nonpregnant women [42]. It is not known if an equivalent immunological response can be expected in pregnant women. These data raise apprehensions because favorable perinatal outcomes depend a great deal upon amplified helper T cell type 2 and regulatory T cell activity coupled with decreased Th1 responses. Alteration of CD4 + T cell responses during pregnancy is related to unfavorable pregnancy outcomes such as preterm birth and fetal loss [43]. Moreover, some evidence suggests that babies born to mothers with variant CD4 + T cell responses may suffer enduring adverse consequences [44].

Until present, the FDA has not issued any explicit guidelines regarding the use of COVID-19 vaccines in pregnant women. Rather, the FDA refers to pregnant women in the EUA letters and factsheets provided to healthcare providers for the individual vaccines. The EUA letters for both the Moderna and the Pfizer/BioNTech vaccines contain provisions obliging post-authorization observational studies and label pregnant women as a “population of interest” for these studies, citing a lack of data regarding vaccine risk in pregnancy. The Moderna vaccine fact sheet specifically alludes to a reproductive toxicity study in female rats; adverse effects on fetal development, female fertility, and early offspring development were assessed, with no adverse outcomes observed. The Moderna vaccine also has a pregnancy exposure registry intended to monitor pregnancy outcomes in women who received the vaccine during pregnancy. To date, neither Moderna nor Pfizer have issued guidelines or guidance regarding their vaccines and pregnancy [41]. The aforementioned concerns notwithstanding, recent data indicate “no difference in the composite primary outcome of preterm birth, preeclampsia with severe features, and cesarean delivery for fetal indication among women with and without SARS-CoV-2 infection diagnosed during pregnancy” (52 women [21 %] vs. 684 women [23 %]; relative risk, 0.94; 95 % CI, 0.73–1.21; *P* = .64). There were also no stillbirths among women with SARS-CoV-2 during pregnancy [45].

### Immunocompromised patients

Current evidence suggests that both the Moderna and the Pfizer/BioNTech vaccines elicit a strong humoral response due to the production of neutralizing antibodies coupled with a robust cellular response by inducing functional and pro-inflammatory CD4 + and CD8 + T cells and expression of Th1 cytokines [46]. Notably, the initial vaccine trials excluded immunocompromised patients, including those on immunosuppressive medications and patients with autoimmune conditions [29, 30]. This population requires special consideration because infections are amongst the most frequent causes of death in them. Notwithstanding, data from the COVID-19 rheumatology registry thus far has not demonstrated an augmented risk of COVID-19 complications in immunocompromised patients with the exception of patients taking moderate or high doses of corticosteroids [47]. On top of the undetermined effectiveness of the COVID-19 vaccine in these patients, there are various other unanswered inquiries regarding vaccinations in patients on immunosuppressive agents [46–48]. Patients on immunosuppressive therapy are known to mount an attenuated immune response to vaccinations and therefore require special consideration for COVID-19 vaccines [42]. Because this patient population was not included in the initial trials, the immunological effectiveness of the mRNA vaccines remains unknown [29, 30]. Healthcare providers may consider holding immunosuppressive therapy for two weeks post-vaccination until future clinical trials will provide further scientific guidance [47]. For both the Moderna and the Pfizer/BioNTech vaccines, the only advisory provided by the FDA for immunocompromised patients is the possibility of moderated response to the vaccine. The CDC states that immunocompromised patients may obtain the vaccines provided they have no contraindications to vaccination, but that they should be advised about the undetermined efficacy and safety profiles of the vaccines in immunocompromised populations [47].

## Discussion

Individual immunological status and circumstantial considerations notwithstanding, the available evidence appears to favor vaccination with either the Moderna or the Pfizer/BioNTech mRNA vaccines for the majority of the population. The vaccination program’s effectiveness depends upon convincing efficacy and safety data coupled with popular public acceptance and inoculation [49]. However, vaccine hesitancy remains a noteworthy challenge in the United States [50]. Much of this hesitancy has stemmed from a relatively abbreviated clinical trial process; despite the swift timeline, patients and providers should bear in mind that these vaccines went through the appropriate due diligence, just like prior vaccines. The CDC also continues to closely monitor these vaccines for safety and efficacy. Furthermore, United States federal government and industry partners had constructed vaccine manufacturing facilities ahead of time prior to vaccine approval. Usually, vaccine manufacturers do not build such facilities until phase 3 has been completed [51].

Vaccine hesitancy has been described as a “lack of confidence in vaccination and/or complacency about vaccination” that may result in deferment or failure to vaccinate in spite of accessible services [50]. The swift vaccine development timelines for the COVID-19 vaccines in particular, combined with the highly divided socio-political landscape, may further compromise vaccination confidence and escalate complacency regarding vaccination [50]. Impressive declines in new-onset COVID-19 infection rates following vaccine dissemination in special populations, e.g. nursing home residents, have highlighted the importance of widespread immunization. Implementation of comprehensive evidence-based efforts targeted at behavior change is necessary to address ongoing vaccine hesitancy. Recent studies reveal vaccine hesitancy and ambivalence in a significant percentage of the public [52]. These surveys further suggest that vaccine hesitancy is more pronounced in populations who have been disproportionately affected by COVID-19, including unemployed individuals and those with lower educational levels, as well as among certain racial minority groups including African Americans and Hispanic Americans [53, 54].

In order to address vaccine hesitancy and enhance COVID-19 vaccine adoption, multi-tiered, evidenced-based approaches must be implemented. They include evidence-based initiatives from behavioral, communication, implementation, and social sciences that can guide clinical programs at the organizational, relational, and individual levels to support public health initiatives and challenge COVID-19 vaccine hesitancy [50]. Important vaccine education platforms include; websites, television, educational programs in school, and vaccine education initiatives to serve areas with limited technological connection. Effective strategies to increase vaccine adoption include implementing vaccination programs for the following: schools and colleges, woman-infant programs, and indigent areas that have less geographic access to vaccination centers [55]. Although it has been noted that children are at lower risk of adverse effects, vaccination among this population remains crucial to limiting the spread of COVID-19 and achieving herd immunity. Healthcare provider endorsement has been shown to result in increased adoption of a variety of preventive healthcare activities including vaccinations. Healthcare professionals are regarded as the most trusted sources of information, generally and specifically regarding COVID-19. Clear and assertive recommendations from healthcare providers may mitigate concerns about safety and enhance vaccine adoption [56]. Evidence suggests that individual-level strategies in the absence of other initiatives remain largely ineffective. However, when used as an adjunct to community-based and interpersonal initiatives, individual approaches can promote vaccination rates and enhance initiatives aimed at reducing hesitancy [57]. Moreover, providers must be aware of the crucial role of the vaccine in the prevention of COVID-19 in the individual and population levels. The development of various vaccines with different dosing schedules and storage needs underscores the need for effective and consistent communication and logistical standards. Healthcare providers must therefore be equipped with training and resources for making strong recommendations and attending to vaccine hesitancy [50]. Development and distribution of consistent, culturally sensitive, and straightforward patient education materials in tandem with other evidence-based strategies can increase vaccination rates [58]. This strategy can be bolstered by drawing upon communication science data, such as positively framed messages and appealing to altruism and prosocial behavior to increase adoption [59].

Notably, a recent survey conducted by the Kaiser Family Foundation found that 29 % of healthcare providers themselves expressed hesitancy about receiving the COVID-19 vaccine. The same survey found that among the general public, the group that reported that they “definitely will not get vaccinated” may be the hardest to reach via most traditional public health means. Only two emissaries were reported as trustworthy sources by at least half the people in this group: their personal health care provider (59 %) and former President Trump (56 %). These findings suggest that individual health care provider endorsement and support may be one of the sole avenues for reaching this group with reliable and timely vaccine information [60].

Practical limitations oblige organizations to choose from the available evidence-based approaches to identify strategies that are achievable and sufficient within their particular circumstances and settings. Initiatives designed to improve uptake of COVID-19 vaccination must therefore be chosen and customized to conform to the specific needs and resources of clinical environments and to tackle recognized obstacles to adoption. Thus, healthcare organizations must assess local contexts in order to appreciate pertinent roadblocks and resources. Moreover, framing initiatives within iterative assessment approaches can aid in effectively identifying and amending the appropriate strategies over time.

## Conclusions

The current data suggests that the currently approved mRNA-based COVID-19 vaccines are safe and effective for the vast majority of the population. Furthermore, broad-based vaccine uptake is critical for achieving herd immunity; an essential factor in decreasing future surges of COVID-19 infections. Ensuring sufficient COVID-19 vaccination adoption by the public will involve attending to the rising vaccine hesitancy among a pandemic-weary population. Evidence-based approaches at the federal, state, city, and organizational levels are necessary to improve vaccination efforts and to decrease hesitancy. Educating the general public about the safety of the current and forthcoming vaccines is of vital consequence to public health and ongoing and future large-scale vaccination initiatives.

## Data Availability

Please contact the authors for data requests.

## Declarations

### FDA clearance

Not applicable (Review).
