# Correction to: The safety of Covid-19 mRNA vaccines: a review

**DOI:** 10.1186/s13037-021-00296-4

**Published:** 2021-05-18

**Authors:** Pratibha Anand, Vincent P. Stahel

**Affiliations:** 1 University of Colorado (CU) School of Medicine, 13001 E 17th Place, Aurora, CO 80045 USA; 2 University of Colorado (CU) Boulder Undergraduate, Program, Boulder, CO 80309 USA

**Correction to: Patient Saf Surg 15, 20 (2021)**

**https://doi.org/10.1186/s13037-021-00291-9**

Following publication of the original article [1], it was noticed that the published version of this article has contained incorrect title. The correct title is "The safety of Covid-19 mRNA vaccines: a review". The original article has been corrected.
